# Exploring the Causality Between Body Mass Index and Sepsis: A Two-Sample Mendelian Randomization Study

**DOI:** 10.3389/ijph.2023.1605548

**Published:** 2023-05-02

**Authors:** Juntao Wang, Yanlan Hu, Jun Zeng, Quan Li, Lanfen He, Wenjie Hao, Xingyue Song, Shijiao Yan, Chuanzhu Lv

**Affiliations:** ^1^ International School of Public Health and One Health, Hainan Medical University, Haikou, Hainan, China; ^2^ Emergency Medicine Center, Sichuan Provincial People’s Hospital, University of Electronic Science and Technology of China, Chengdu, China; ^3^ Emergency Department, State Key Laboratory of Complex Severe and Rare Diseases, Peking Union Medical College Hospital, Chinese Academy of Medical Science and Peking Union Medical College, Beijing, China; ^4^ Key Laboratory of Emergency and Trauma of Ministry of Education, Hainan Medical University, Haikou, China; ^5^ Department of Emergency, Hainan Clinical Research Center for Acute and Critical Diseases, The Second Affiliated Hospital of Hainan Medical University, Haikou, Hainan, China; ^6^ Research Unit of Island Emergency Medicine, Chinese Academy of Medical Sciences (No. 2019RU013), Hainan Medical University, Haikou, China

**Keywords:** obesity, body mass index, Mendelian randomization, sepsis, instrumental variable

## Abstract

**Objective:** Observational epidemiological studies have shown a link between obesity and sepsis, but any causal relationship is not clear. Our study aimed to explore the correlation and causal relationship between body mass index and sepsis by a two-sample Mendelian randomization (MR).

**Methods:** In large sample genome-wide association studies, single-nucleotide polymorphisms related to body mass index were screened as instrumental variables. Three MR methods, MR-Egger regression, weighted median estimator, and inverse variance-weighted, were used to evaluate the causal relationship between body mass index and sepsis. Odds ratio (OR) and 95% confidence interval (CI) were used as the evaluation index of causality, and sensitivity analyses were conducted to assess pleiotropy and instrument validity.

**Results:** By two-sample MR, the inverse variance weighting method results suggested that increased body mass index was associated with an increased risk of sepsis (odds ratio 1.32; 95% CI 1.21–1.44; *p* = 1.37 × 10^−9^) and streptococcal septicemia (OR 1.46; 95% CI 1.11–1.91; *p* = 0.007), but there was no causal relationship with puerperal sepsis (OR, 1.06; 95% CI, 0.87–1.28; *p* = 0.577). Sensitivity analysis was consistent with the results, and there was no heterogeneity and level of pleiotropy.

**Conclusion:** Our study supports a causal relationship between body mass index and sepsis. Proper control of body mass index may prevent sepsis.

## Introduction

Sepsis is a life-threatening organ dysfunction caused by a dysregulated host response to infection (1). A systematic review inferred that there are about 30 million sepsis episodes and 6 million deaths worldwide every year (2). Sepsis often leads to the functional involvement of multiple organs in the whole body and induces various complications. Its clinical severity and outcome depend on the location and type of infection, individual response to inflammation, and treatment measures (3). Therefore, improving the prevention, diagnosis, and management of sepsis is of great significance in reducing the global disease burden.

Body mass index (BMI) is an index of the degree of obesity and health. In recent years, it has often been used to predict the risk of related diseases. Observational studies have found an association between BMI and sepsis. High BMI has a protective effect on the mortality of sepsis, but morbid obesity and underweight have nothing to do with the reduction of mortality (4). Due to the influence of confounding factors between exposure and outcome, the causal relationship between BMI and sepsis needs to be further explored.

Mendelian randomization (MR) detects and quantifies the causal relationship between exposure and disease by using genetic variation as an instrumental variable (IV) (5). With the publication of a large amount of exposure and disease-related genetic variation data in genome-wide association studies (GWAS) (6), MR research has made a breakthrough. Compared with observational studies, MR studies using genetic variation as a tool can control for unmeasured confounders and reverse causality (7), which are now mostly used for causal inference of etiology.

## Methods

### Study Design

**FIGURE 1:**
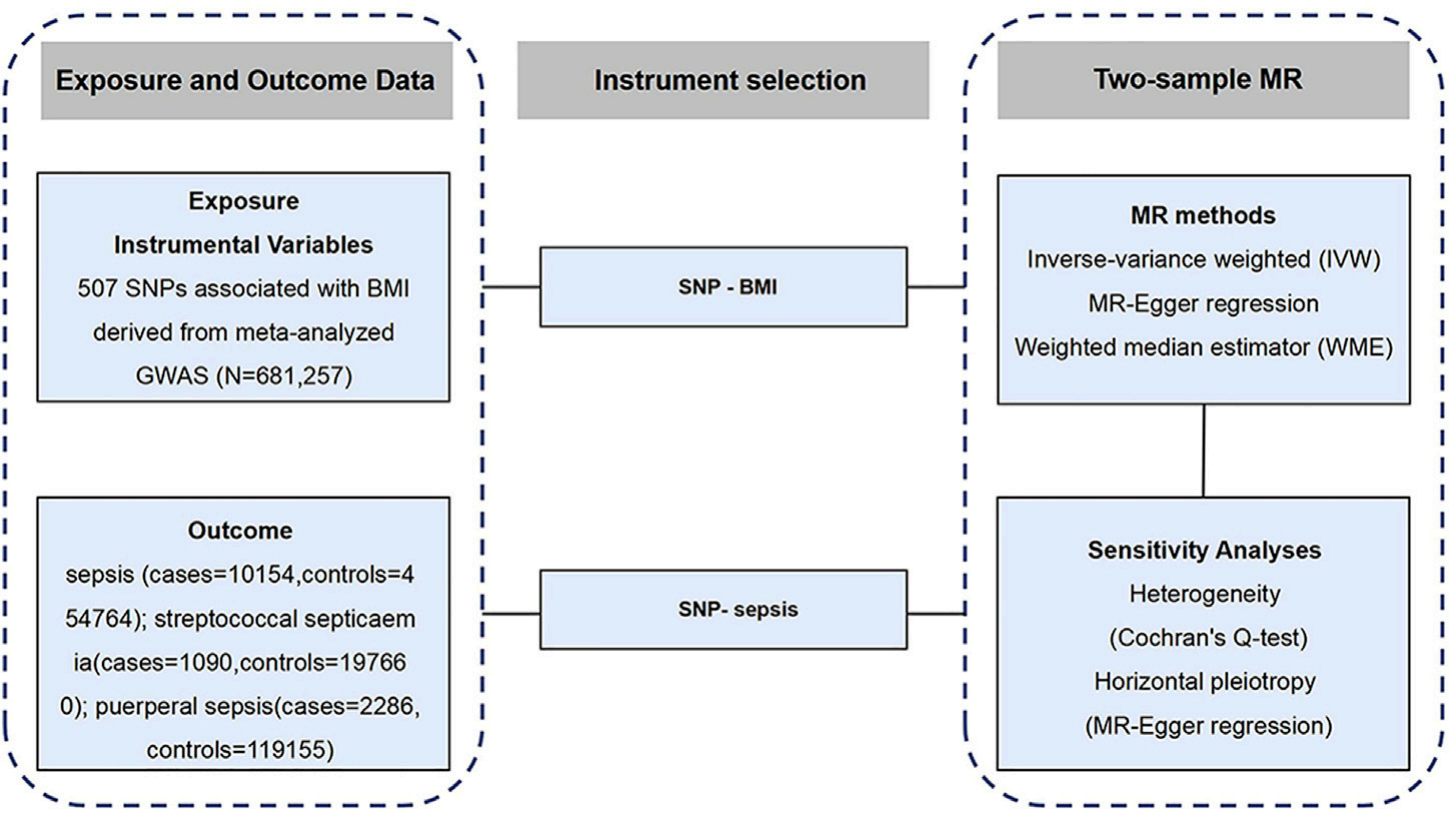
Schematic representation of the study design (Exploring the Causality Between Body Mass Index and Sepsis: A Two-Sample Mendelian Randomization Study, China, 2023).

We designed a two-sample Mendelian randomization (2SMR) study to explore the causal association between BMI and sepsis (Figure 1). Firstly, the complete summary data set was obtained from the Genetic Investigation of Anthropologic Traits (GIANT) alliance and IEU OpenGWAS open-source database, and the single-nucleotide polymorphisms (SNPs) directly related to BMI were selected as the IV. Secondly, three MR methods, inverse variance-weighted (IVW), weighted median estimator (WME), and MR-Egger regression, were used to analyze the causal relationship. Finally, sensitivity analysis was performed. Cochran’s Q-text was used to evaluate the heterogeneity of MR results, and the level of pleiotropy was determined by MR-Egger regression.

### Source and Acquisition of GWAS

The data on BMI were extracted from the meta-analysis of a large GWAS. The database is based on the public database of GIANT alliance. The GWAS results related to BMI in the database were published on human molecular genetics in 2018 (8). The sample size of the study was 681,257 people, and 2,336,260 SNP sites related to BMI were analyzed. The data download website is https://portals.broadinstitute.org/collaboration/giant/index.php/GIANT_consortium_data_files.

The GWAS data of sepsis came from the IEU OpenGWAS database. A total of 10,154 cases and 454,764 controls were included in the study, all of European descent, consistent with the exposure data. The website for data download is https://gwas.mrcieu.ac.uk/. In addition, GWAS data for streptococcal septicemia and puerperal sepsis were obtained from the FinnGen cohort, which included 96,499 participants and was available from the MR-Base website http://www.mrbase.org/ (9). Sepsis is defined according to the third international consensus on sepsis and septic shock published by the American Medical Association in 2016 (1). All GWAS data on the included studies are available in Supplementary Table S1.

### IVs

**FIGURE 2:**
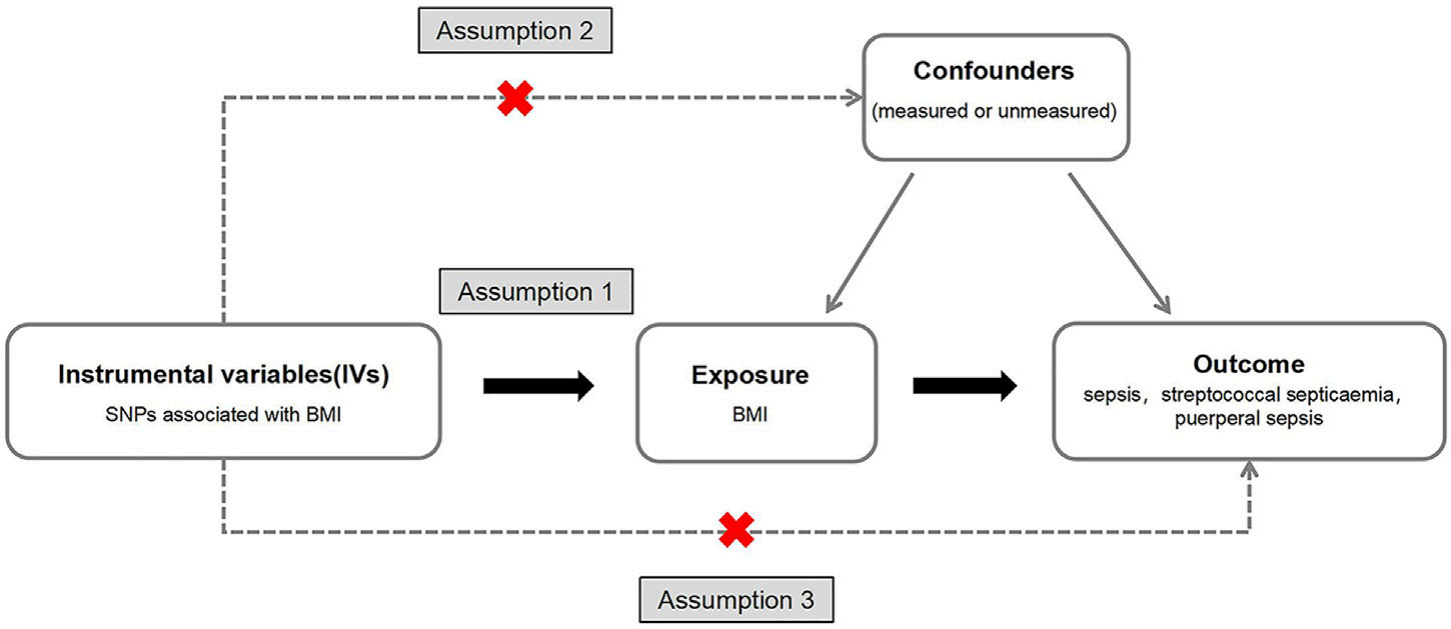
A directed acyclic graph of Mendelian random research design. We selected body mass index related single nucleotide polymorphisms as a genetic tool to assess whether body mass index has a causal effect on sepsis. In order to make a causal inference about the impact of body mass index on sepsis, we must also assume that the selected single nucleotide polymorphisms are not related to confounders or outcomes (Exploring the Causality Between Body Mass Index and Sepsis: A Two-Sample Mendelian Randomization Study, China, 2023).

The SNPs associated with BMI were screened as IVs: (1) SNPs has the significance of genome-wide study (*p* < 5 × 10^−8^); (2) The SNPs were kept independent of each other, and the SNPs in linkage disequilibrium were excluded, with R^2^ > 0.001 and distance located 10,000 kb apart from each other. The MR had three requirements (Figure 2):

(1) IVs have a strong correlation with exposure factors. If the correlation between IVs and exposure is weak, it is easy to produce weak IV bias. It is generally believed that there is no weak IV bias when F > 10, where $F=\frac{R^{2}\left(N-2\right)}{1-R^{2}}$ , R^2^ is the proportion of variation explained by SNPs in exposure data, and N is the sample size in exposure database (5). The R^2^ is calculated as follows: $R^{2}=\frac{2\times EAF\times \left(1-EAF\right)\times {beta}^{2}}{{SD}^{2}}$ , where EAF is the effector allele frequency, beta is the allele effect value, and SD is the standard deviation (10) (Supplementary Table S2).
(2) IVs are not associated with confounding factors. Genotype should not be associated with confounders in MR—this hypothesis is often difficult to directly prove due to the lack of individual level data. The validity of associations between IV and confounders is sometimes demonstrated using long-accepted criteria in observational epidemiology (11).
(3) IVs have no direct correlation with outcome variables, and can only be related to outcomes through exposure.

Pleiotropy occurs when a genetic locus affects multiple traits (12). Horizontal pleiotropy can distort the causal estimation of MR and increase the type I error rate (false positive) (13).

### Statistical Analysis

In our MR study, IVW was the main statistical method used to test the causal association between BMI and sepsis risk. Results were expressed as the odds ratio (OR) and 95% confidence interval (CI) for the risk of sepsis for each 1 SD increase in BMI. In addition, sensitivity analysis was used to verify the robustness of MR results, including MR-Egger regression to evaluate the bias caused by invalid IVs and pleiotropic IVs, whose intercept term was 0 (*p* < 0.05), indicating that there was no gene pleiotropy (14). When the effective IVs exceed 50%, the WME method can obtain consistent causal effect estimation (15). Heterogeneity between IVs was assessed using the Cochranʼs Q statistic, with *p* < 0.05 indicating heterogeneity, which could be controlled by the random-effects IVW.

The above statistical procedures were performed in R software (version 4.1.2) using TwoSampleMR (16) software packages. In addition, the MR-Base platform was used to draw Leave-one-out, scatter, and forest plots of BMI and sepsis-related SNPs to visualize the statistical analysis results, indicating the effectiveness of MR analysis. The test level was a = 0.05, and *p* < 0.05 was considered significant.

## Results

### IV Selection for BMI

Details of the SNPS associated with BMI are shown in Supplementary Table S2. After screening, 507 SNPs closely related to BIM were identified as IVs. After reconciling exposure and outcome, 13 palindromic alleles with intermediate frequencies were removed for 2SMR. All SNPs obtained were strong IVs, and F values (range 28–1,426) were all greater than the recommended threshold of 10, indicating that there was no bias caused by weak IVs in the study. Online tools (https://shiny.cnsgenomics.com/mRnd/) were used to calculate each SNPs’ MR analysis of statistical power of sepsis (17).

### Mendelian Randomization Between BMI and Sepsis

**FIGURE 3:**
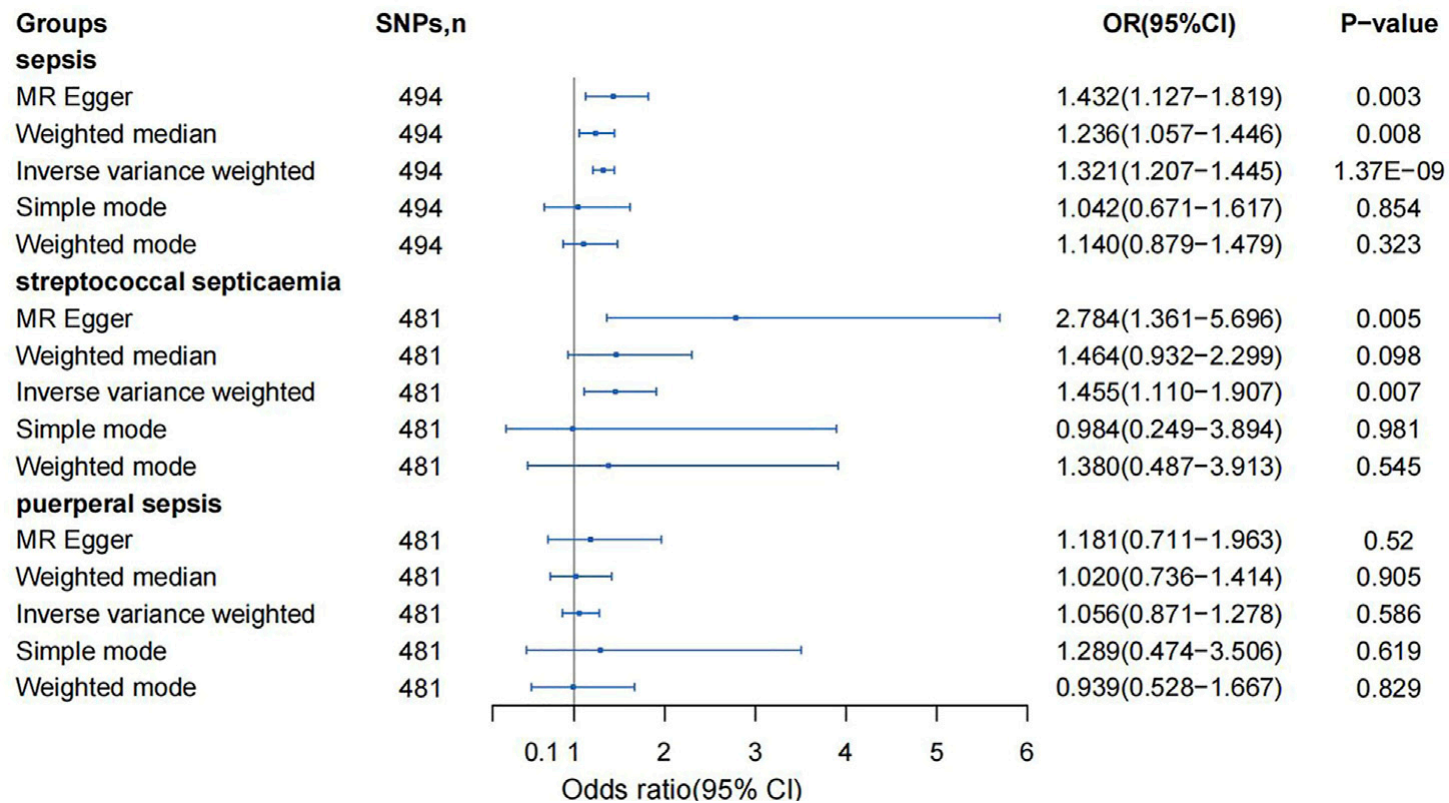
Mendelian randomization analysis on the association between body mass index and sepsis (Exploring the Causality Between Body Mass Index and Sepsis: A Two-Sample Mendelian Randomization Study, China, 2023).

**FIGURE 4:**
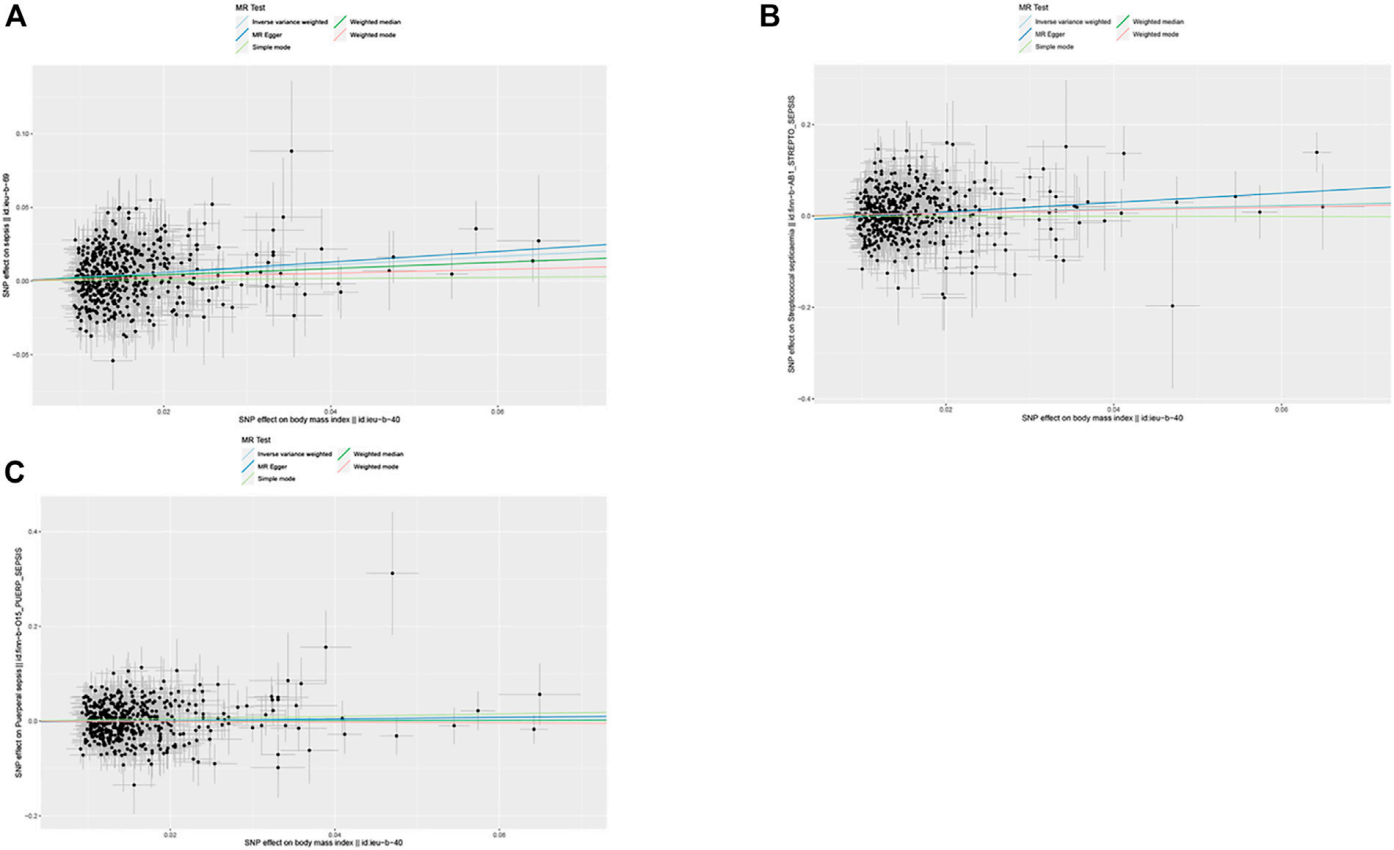
Scatter plots of single nucleotide polymorphism effect on body mass index and single nucleotide polymorphism effect on sepsis. **(A)** Sepsis, **(B)** streptococcal septicemia, **(C)** puerperal sepsis (Exploring the Causality Between Body Mass Index and Sepsis: A Two-Sample Mendelian Randomization Study, China, 2023).

The IVW method was used for causal analysis, and the results supported the association of genetically predicted BMI with increased risk of sepsis (OR = 1.32, 95% CI = 1.21–1.45, *p* = 1.37 × 10^−9^). In sensitivity analysis, Cochran’s Q-test showed no heterogeneity (Cochran’s Q Statistic = 526.30, *p* = 0.145), and MR-Egger regression analysis showed no pleiotropy (intercept = −0.001; *p* = 0.474). The causal associations using five different methodological methods (IVW, MR-Egger, Simple Mode, WME, and Weighted Mode) are shown in Table 1. Forest plots showed the causal effect of BMI on sepsis risk (Figure 3). The scatter plot illustrates the causal relationship between BMI and sepsis risk (Figure 4). The leave-one-out plots illustrated that no single SNPs influenced the estimate of the outcome (Supplementary Figure S1).

**TABLE 1:** Mendelian randomization results of the causal effect of body mass index on the risk of sepsis (Exploring the Causality Between Body Mass Index and Sepsis: A Two-Sample Mendelian Randomization Study, China, 2023).

Outcome	nsnp	Method	Beta	SE	OR (95% CI)	*p*-value	Cochran’s Q statistic (*p*-value)	Egger intercept (*p*-value)
Sepsis	494	MR Egger	0.359	0.122	1.432 (1.127–1.819)	0.003	525.7469 (0.142)	−0.001 (0.474)
		Weighted median	0.212	0.08	1.236 (1.057–1.446)	0.008		
		Inverse variance weighted	0.278	0.046	1.321 (1.207–1.445)	1.37 × 10^−9^	526.2954 (0.145)	
		Simple mode	0.041	0.224	1.042 (0.671–1.617)	0.854		
		Weighted mode	0.131	0.133	1.140 (0.879–1.479)	0.323		
Streptococcal septicaemia	481	MR Egger	1.024	0.365	2.784 (1.361–5.696)	0.005	439.2610 (0.903)	−0.011 (0.056)
		Weighted median	0.381	0.23	1.464 (0.932–2.299)	0.098		
		Inverse variance weighted	0.375	0.138	1.455 (1.110–1.907)	0.007	442.9417 (0.886)	
		Simple mode	−0.016	0.702	0.984 (0.249–3.894)	0.981		
		Weighted mode	0.322	0.532	1.380 (0.487–3.913)	0.545		
Puerperal sepsis	481	MR Egger	0.167	0.259	1.181 (0.711–1.963)	0.520	494.8031 (0.299)	−0.002 (0.637)
		Weighted median	0.02	0.167	1.020 (0.736–1.414)	0.905		
		Inverse variance weighted	0.053	0.098	1.056 (0.871–1.278)	0.586	495.0341 (0.308)	
		Simple mode	0.254	0.51	1.289 (0.474–3.506)	0.619		
		Weighted mode	−0.063	0.293	0.939 (0.528–1.667)	0.829		

### MR Between BMI and Streptococcal Sepsis

The MR analysis was performed using 481 SNPs. The IVW results showed a causal relationship between BMI and streptococcal sepsis (OR = 1.46, 95% CI = 1.11–1.91, *p* = 0.007), without significant heterogeneity (Cochran’s Q Statistic = 442.94, *p* = 0.886). MR-Egger regression analysis showed no significant difference between the intercept and 0, and there was no pleiotropy (intercept = −0.011; *p* = 0.056).

### MR Between BMI and Puerperal Sepsis

The 2SMR results showed no causal relationship between BMI and postpartum sepsis risk (IVW, OR = 1.06, 95% CI = 0.87–1.28, *p* = 0.577; MR-Egger, OR = 1.18, 95% CI = 0.71–1.96, *p* = 0.523), heterogeneity (Cochran’s Q Statistic = 493.56, *p* = 0.325) and pleiotropy (intercept = −0.002; *p* = 0.645).

## Discussion

We designed a 2SMR study to assess whether human genetic evidence supports a causal association between BMI and sepsis (or sepsis). The results showed that elevated BMI was associated with an increased risk of sepsis and streptococcal sepsis, but not postpartum sepsis. This finding was consistent across different analytical methods, and the association between BMI and sepsis was considered robust when combined with a series of sensitivity analyses.

Sepsis is a common and frequently occurring disease in hospital emergency departments, and is one of the major problems affecting human health. High BMI may induce sepsis through some pathophysiological mechanism (18). Early studies suggested that obesity can induce production of proinflammatory cytokines such as tumor necrosis factor (TNF-a) in adipose tissue, leading to inflammatory response in patients with sepsis (19). These inflammatory processes involve the upregulation of systemic immunity, which is clinically manifest as insulin resistance and metabolic syndrome, increasing the risk of organ failure, infectious disease and death in patients (20). The second possible explanation is that the process of oxidative stress is significantly worsened in obese patients with sepsis (21). An animal experiment showed that obese animals were more likely to suffer from the harmful effects of sepsis on peripheral organs due to oxidative damage and low antioxidant levels of enzymes (22).

Observational epidemiological studies have confirmed the association between BMI and sepsis. In a retrospective cohort analysis of 834 patients, among septic patients admitted to the ICU, obese patients were associated with higher mortality than nonobese patients (23). Due to the dysfunction of the immune system in obese people, the prevalence and mortality of sepsis are significantly increased (24). However, this association is not robust, and some studies suggest the exact opposite. Mica et al. believe that high BMI has a protective effect on sepsis (25), which may be related to the increase of leptin in obese people (26). Confusion in causal inferences in observational studies can lead to completely opposite conclusions. Therefore, in this study, we evaluated the causal effect estimates between exposure factors and outcome variables by MR studies and using genetic IV.

Although studies have shown some association between BMI and sepsis, correlation is not equal to causality, and exploring relevant but non-causal influencing factors is not ideal for the prevention and treatment of sepsis. Therefore, understanding the genetic role of sepsis is the key to explaining its pathogenesis and proposing preventive strategies. A recently published MR study associated increased BMI with increased sepsis mortality at 28 days, although the effect disappeared at 90 days (27). In another MR study on BMI and infection risk, high BMI was associated with an increased risk of sepsis (28). Our results supported these two previous studies.

In addition, our study showed that high BMI increased the risk of streptococcal sepsis. Obesity is a risk factor for increased mortality in patients with bacteremia, and bacteremia is one cause of sepsis (29). To our knowledge, this is the first MR study to investigate the association between BMI and the risk of postpartum sepsis. Retrospective observational studies have found that after controlling for the mode of delivery, obese women are twice as likely as normal-weight women to develop the complication of sepsis (30). However, our study showed no causal link, which may be due to the increased incidence and mortality of sepsis during puerperium due to maternal infection with streptococci (30).

We used large samples of GWAS data and detailed validation of MR hypotheses to improve the efficiency of MR analysis. The GWAS data on exposure and outcomes were obtained from individuals of European ancestry, reducing the results of the study by factors such as population stratification. We used several analytical methods to assess the possibility of pleiotropy and showed that the results of WME and MR-Egger regression were similar to those of IVW. More IVs, more power, more statistical methods, and heterogeneity-testing methods can improve the robustness of MR results and prevent reverse causality bias.

There are some limitations to our study. First, when we investigated whether there was a causal relationship between obesity and sepsis, we used BMI, which represents total body obesity, rather than the waist circumference metric used to assess centripetal obesity, despite observational studies concluding that abdominal obesity is also associated with an increased risk of death from sepsis (21). Second, while statistically significant, our study had only a 32% increase in the risk of sepsis for a genetically induced one-unit increase in BMI. Third, because BMI is a highly polygenic trait, pleiotropy cannot be entirely ruled out. Some studies have observed a positive correlation between BMI and inflammatory biomarkers, and excessive inflammation is thought to be the root cause of early organ dysfunction in sepsis (31). As a result, more research is needed to determine whether inflammatory markers mediate the causal relationship between BMI and sepsis. Fourth, our study design was not stratified for BMI, and there is still a lack of data for a more detailed assessment of the “obesity paradox” (20) that may arise from extreme BMI. Fifth, the GWAS included were from European populations. Therefore, considering the differences in body size between European, North American, and Asian people, there may be limitations in the generalization of the results to other populations.

### Conclusion

In conclusion, high BMI was associated with an increased risk of sepsis and streptococcal sepsis, independent of the occurrence of postpartum sepsis. In contrast to observational studies, MR analyses used genetic IVs to estimate the effect of exposure on disease incidence, and our findings further support the finding that appropriate management of BMI may prevent the development of sepsis, a finding with important public health implications.

## Supplementary Material

Supplementary Table S1 Descriptive characteristics of the GWAS meta-analyses used in the current Mendelian randomization study.

Supplementary Table S2 Characteristics of the genetic variants associated with sepsis.

Supplementary Figure S1 Leave-one-out plot. **(A)** Sepsis, **(B)** streptococcal septicemia, **(C)** puerperal sepsis.

## Abbreviations

BMI, Body mass index; MR, Mendelian randomization; GWAS, Genomic-wide association study; SNP, Single-nucleotide polymorphism; IV, Instrumental variable; 2SMR, Two-sample Mendelian randomization; IVW, Inverse variance-weighted; MR-Egger, Mendelian randomization-Egger regression; WME, Weighted median estimator; OR, Odds ratio; SD, Standard deviation; TNF-α, Tumor necrosis factor-alpha.
