# Supplementary figures and images for: Exploring the Causality Between Body Mass Index and Sepsis: A Two-Sample Mendelian Randomization Study
Source: Int J Public Health. 2023 May 2;68:1605548. doi: 10.3389/ijph.2023.1605548 (PMC10186272; doi:10.3389/ijph.2023.1605548)

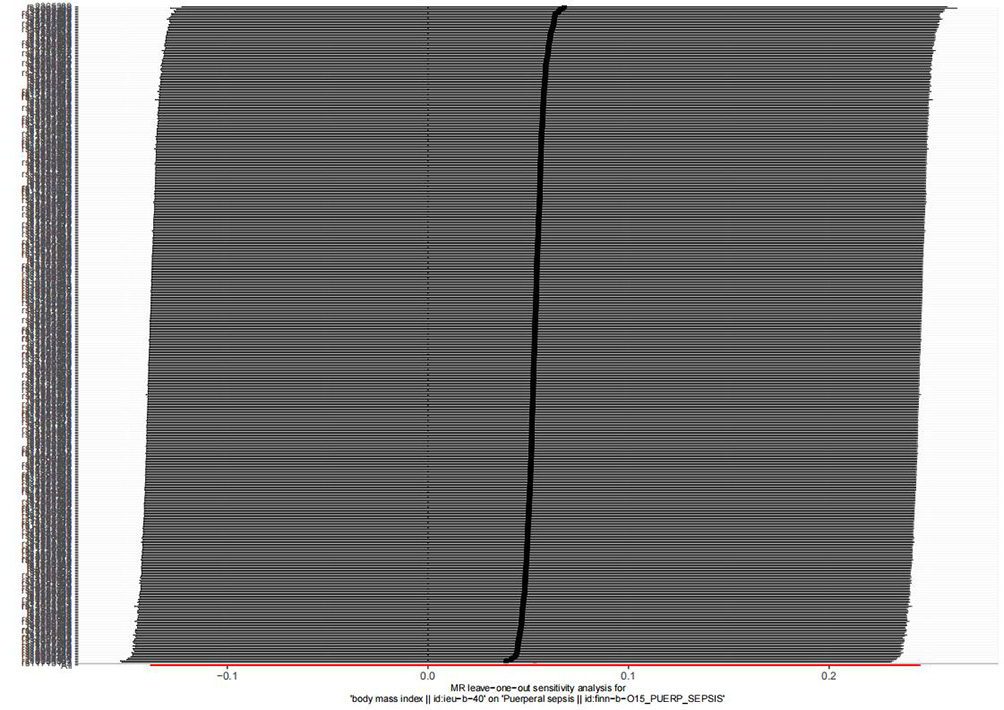

Supplement: Supplementary file 2 [file Image3.JPEG]

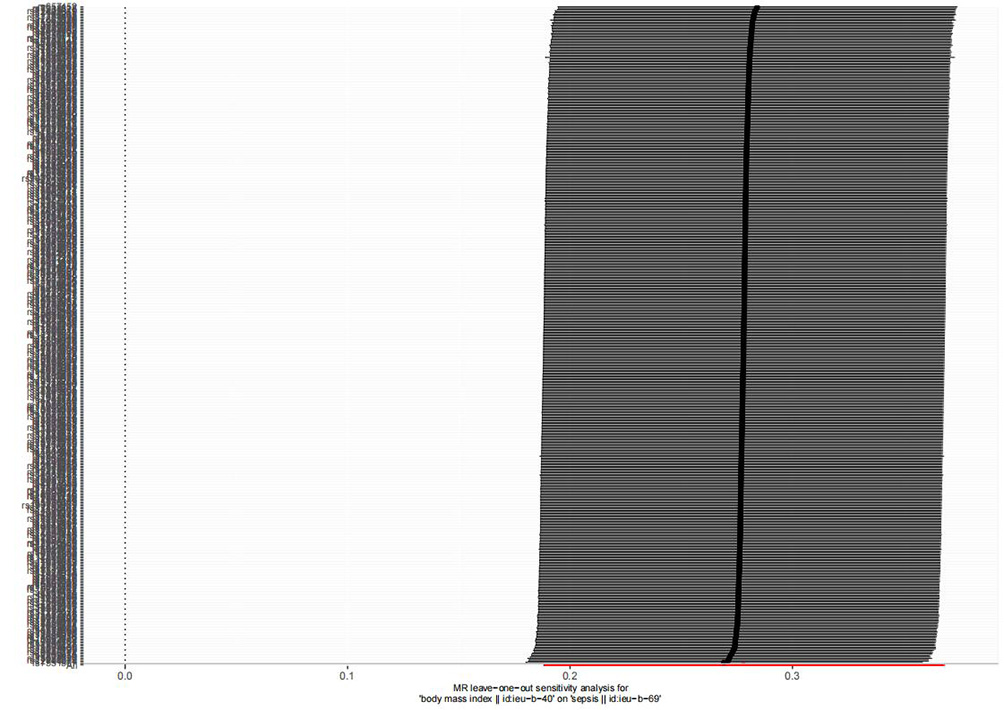

Supplement: Supplementary file 3 [file Image1.JPEG]

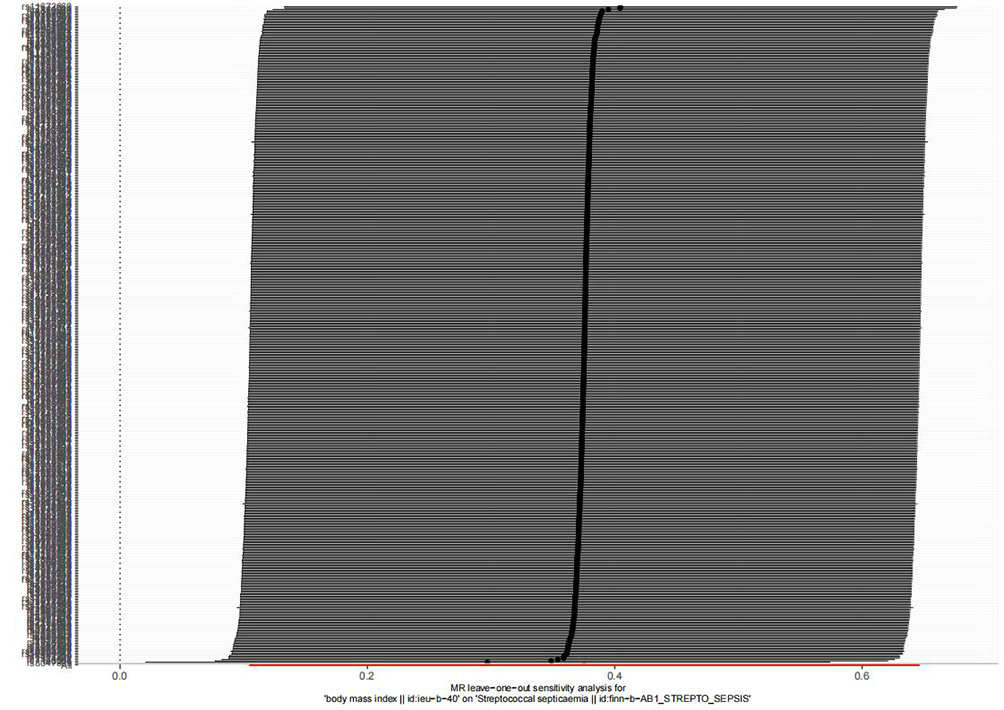

Supplement: Supplementary file 4 [file Image2.JPEG]
